# Corneal neurotization meets COVID-19: a case report of minimally invasive corneal neurotization complicated by COVID-19-related keratitis

**DOI:** 10.1186/s12348-025-00521-6

**Published:** 2025-08-09

**Authors:** Shuqin Zhuang, Qiaoran Qi, Jiaying Zhang, Jin Li

**Affiliations:** 1 Department of Ophthalmology, Shanghai Ninth People’s Hospital, Shanghai Jiao Tong University School of Medicine, No.639, Zhizaoju Road, Huangpu District, Shanghai, Shanghai 200011 China; 2 Shanghai Key Laboratory of Orbital Diseases and Ocular Oncology, Shanghai, China

**Keywords:** Neurotrophic keratopathy, Corneal neurotization, SARS-CoV-2 eye infection, Viral keratitis, Corneal nerves

## Abstract

**Purpose:**

To report a rare case of severe keratitis followed by SARS-CoV-2 infection after minimally invasive corneal neurotization (MICN) surgery for the first time.

**Methods:**

Retrospective single case report.

**Results:**

A 59-year-old female presented to ophthalmology department of our hospital with facial paralysis induced by neurosurgeries. After detailed ophthalmological examination, she was diagnosed with Mackie stage III neurotrophic keratopathy (NK) in the right eye and subsequently underwent MICN and amniotic membrane transplantation. Postoperatively, corneal sensation and the epithelial defect showed gradual improvement, with corneal sensation recovering to 30 mm (measured by Cochet-Bonnet esthesiometer). However, thirteen months after MICN, she developed a corneal ulcer in the right eye one week after contracting SARS-CoV-2 (COVID-19). Supplementary investigations, including anterior segment photography, in vivo confocal microscopy (IVCM), and corneal scraping for pathogen detection, revealed recurrent corneal anesthesia and loss of corneal nerves, with no pathogens identified. After two weeks of empirical antiviral and antibacterial therapy yielded no significant improvement, a diagnosis of COVID-19-induced neurotrophic keratitis (Stage III Mackie) was established. Management consisted of prolonged medical therapy (including antiviral agents, antibiotics, nutritional supplementation, corticosteroids, and artificial tears), two amniotic membrane transplants, and one temporary tarsorrhaphy. Following two years of treatment and follow-up, the corneal lesion achieved complete healing with corneal nerve regeneration and restoration of corneal sensation.

**Conclusion:**

This study presents the first documented case of COVID-19-related NK following MICN established as a diagnosis of exclusion. This case underscores the critical need for comprehensive differential diagnosis to rule out infectious etiologies in post-MICN keratitis, ultimately leading to a diagnosis of exclusion for COVID-19-induced disease. The diagnostic approach outlined may offer valuable insights for similar presentations. After a protracted clinical course, the patient ultimately achieved restoration of corneal sensation and reinnervation, demonstrating the preserved regenerative potential of MICN-reconstructed neural pathways even after severe viral infection.

**Supplementary Information:**

The online version contains supplementary material available at 10.1186/s12348-025-00521-6.

## Introduction

Neurotrophic keratopathy (NK) is a refractory degenerative disease resulting from trigeminal nerve impairment caused by surgery, trauma, herpes virus infection or systemic conditions such as diabetes mellitus, manifesting corneal sensation loss due to nerve injury [1]. Currently, the primary therapeutic approach involves the administration of recombinant human nerve growth factor [1]. In cases of trigeminal nerve injury following cranial neurosurgery, prolonged treatment is often necessary. Despite its therapeutic promise, real-world application faces dual challenges of unsustainable cost structures. As an innovative surgical solution, minimally invasive corneal neurotization (MICN) mediates corneal reinnervation through microsurgical transfer of sural nerve segments, providing both mechanosensory recovery and crucial neurotrophic support to counteract the disease process in NK [2–4]. The intervention carries predictable risks of corneal neovascularization and transient sensory impairment in the donor nerve territory.

To date, there is no documented evidence of viral keratitis after MICN. Viral damage to corneal nerves impairs the regenerative process of NK post-MICN. Here, we present the inaugural case of severe NK complicated by COVID-19-related keratitis subsequent to MICN. After a protracted two-year treatment course, the patient ultimately achieved restoration of corneal nerve and sensation.

## Case presentation

### Initial visit

In December 2021, a 59-year-old woman presented to Shanghai Ninth People’s Hospital with a 1.5-year history of visual acuity decline in her right eye. She had a prior history of resection of a right-sided acoustic neuroma at an external medical facility, complicated by right facial paralysis (House-Brackmann Grade VI) and NK (Mackie stage III). She had undergone right eyelid tarsorrhaphy fifteen months earlier and facial nerve transplantation with nerve anastomosis four months prior. She denied any history of diabetes mellitus, AIDS, or autoimmune conditions such as rheumatoid arthritis.

On examination, the right eye demonstrated a central corneal lesion characterized by epithelial erosion, stromal edema and thickening, and neovascularization. The best-corrected visual acuity (BCVA) was 0.01, and corneal sensation was 0/60 using the Cochet-Bonnet esthesiometer (Luneau Ophtalmologie, Chartres, France). The Cochet-Bonnet esthesiometer stimulates mechanoreceptors and polymodal nociceptors, which constitute approximately 90% of corneal nociceptors [5]. Corneal sensation testing was conducted by the same examiner. During the assessment, the patient was seated indoors under natural lighting, initially fixating straight ahead. The examiner applied perpendicular pressure to the central cornea, starting with a filament length of 60 mm and decreasing in 5 mm increments. The maximum filament length eliciting a positive response was recorded as the corneal sensitivity threshold, with three consecutive verifications [6]. Subsequently, she was instructed to sequentially gaze into each of the four peripheral quadrants to evaluate corneal sensation in each region. Results were documented in millimeters of filament length.

In vivo confocal microscopy (IVCM) examination was conducted utilizing the Rostock Cornea Module of the Heidelberg Retina Tomograph II (HRT II RCM, Heidelberg Engineering GmbH, Heidelberg, Germany). IVCM demonstrated a significant reduction in subepithelial nerve fibers, with most regions lacking nerve innervation. She was diagnosed with Mackie stage III NK. Given the refractory nature of her NK and inadequate response to conventional therapy, she was indicated for corneal neurotization.

### Minimally invasive corneal neurotization

Corneal neurotization procedures are primarily categorized into direct corneal neurotization (DCN) and indirect corneal neurotization, known as MICN [7, 8]. In DCN, the supratrochlear and supraorbital nerves are identified via a coronal incision at the vertex and subsequently dissected [9, 10]. The principle of MICN involves anastomosing the contralateral supraorbital nerve and supratrochlear nerve with a free sural nerve graft through a minimally invasive approach [11, 12]. Approximately 12–14 cm of sural nerve is harvested as the donor nerve [11, 12]. A 2-cm incision is made in the upper eyelid to isolate the nerve branches of the trochlear and supraorbital nerves [12, 13]. The choice of surgical technique depends on the patient’s condition, the extent of trigeminal nerve damage and the availability of donor nerves. The patient had previously undergone craniotomy for acoustic neuroma resection, and DCN could potentially increase the risk of complications such as encephalitis [10]. In DCN, dissection of the supraorbital nerve may result in forehead hypoesthesia. A section of sural nerve was easy to obtain as the donor nerve in this case. Our hospital was the first medical center in China to perform MICN, with the largest case volume to date. Given that both approaches are accepted treatments for NK with comparable clinical outcomes at twelve months, MICN was considered to cause less trigeminal nerve dysfunction and was deemed safer and technically feasible for this patient without compromising therapeutic goals [10]. Thus, she timely underwent MICN and amniotic membrane transplantation (AMT).

**Fig. 1:**
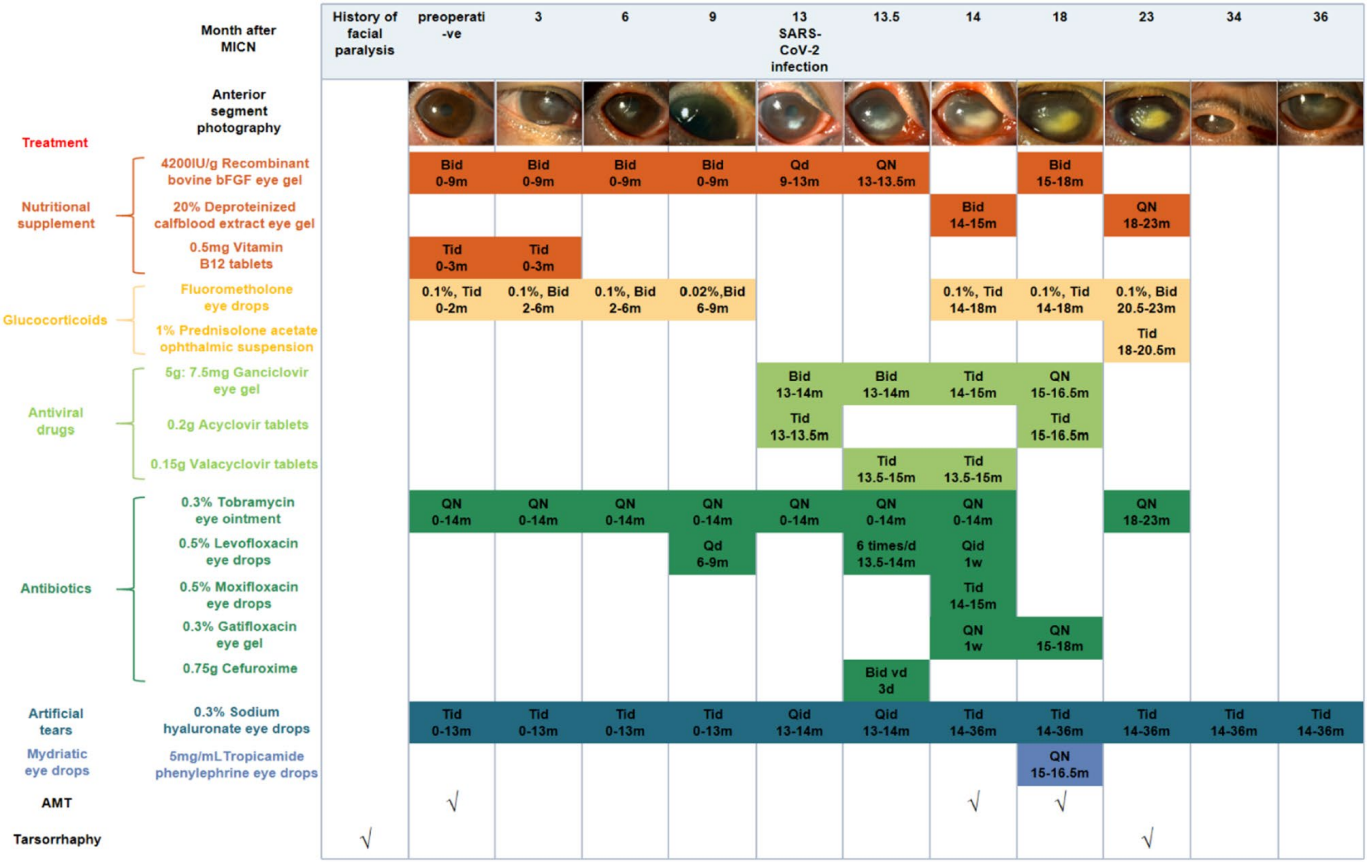
Pictures of anterior segment photography and treatment of the patient. Abbreviations: bFGF, basic fibroblast growth factor

Serial examinations at one to three months post-MICN demonstrated gradual but significant improvement in corneal epithelial integrity and stromal clarity (Fig. 1). Corneal sensation in the temporal quadrant improved to 5/60 (Table 1). IVCM revealed elongated, continuous nerve fibers with improved longitudinal trajectories, suggesting nerve regeneration (Table 2). Corneal nerve fiber length (CNFL) refers to the total length of corneal nerves per mm^2^.Five representative images of the central corneal sub-basal nerve plexus and dendritic cells (DCs) were selected for quantitative analysis based on criteria including the complete image of the same layer, optimal contrast and maximal visibility of nerve fibers and DCs. Sub-basal nerve plexus analysis was performed using an automated software ACCMetrics (MA Dabbah, Imaging Science and Biomedical Engineering). DC density was calculated as the total number of DCs per image and was analyzed using ImageJ. Postoperative corneal sensation and nerve density gradually increased. During the first three postoperative quarters (nine months) following MICN, her corneal epithelium, sensation and nerves gradually recovered (Tables 1 and 2).

**Table 1:** The corneal sensation of the patient

Time	Central cornea	Upper quadrant	Lower quadrant	Nasal quadrant	Temporal quadrant
pre	0	0	0	0	0
post 3 m	0	0	0	0	5
post 6 m	5	25	10	20	30
post 9 m	10	15	10	20	30
post 34 m	20	30	20	25	40

**Table 2:** Changes in the in vivo confocal microscopy outcomes of the patient

Variables	pre	post 3 m	post 6 m	post 9 m	post 13 m	post 13.5 m	post 14 m	post 36 m
CNFL (mm/mm^2^)	5.46 ± 2.43	9.46 ± 2.31	11.47 ± 4.62	15.09 ± 1.07	6.15 ± 1.30	2.55 ± 0.87	4.50 ± 1.02	9.08 ± 2.96
DC (no./frame)	9.20 ± 5.22	16.20 ± 2.78	7.00 ± 3.16	3.80 ± 1.10	7.20 ± 2.95	41.80 ± 26.13	88.60 ± 20.55	6.60 ± 2.07

Postoperative care included: nutritional supplementation (corneal nerve regeneration), glucocorticoids (inflammation control/immune rejection prophylaxis), nocturnal antibiotic ointment (exposure/infection prevention), and artificial tears (adjunctive therapy) (Fig. 1). Low-concentration glucocorticoids were withdrawn at nine months post-surgery.

### SARS-CoV-2 infection episode

**Fig. 2:**
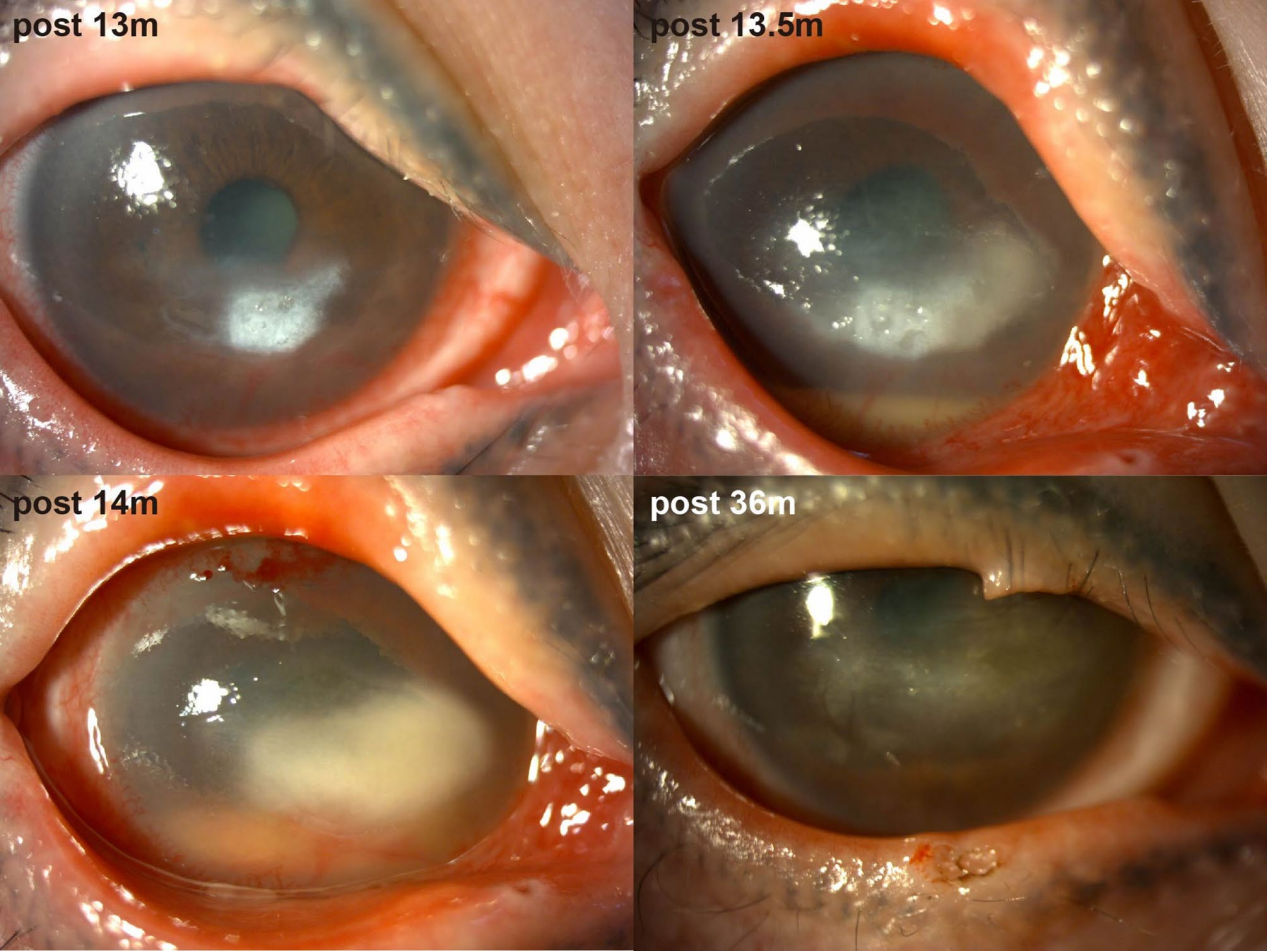
Pictures of anterior segment photography of the patient postoperatively after SARS-CoV-2 infection

Thirteen months post-MICN, the patient developed COVID-19 confirmed by antigen testing, manifesting as a self-limited upper respiratory tract infection. Within one week of viral onset, she experienced acute visual deterioration, representing disease recurrence. Ocular examination revealed a white infiltrative corneal lesion adjacent to the limbus at the 4–5 o’clock position in the right eye, characterized by well-demarcated borders, epithelial defect, stromal edema, and increased purulent secretion (Fig. 2). IVCM revealed inflammatory cell infiltration in the lesion area, and no fungal hyphae or other pathogens were detected. Based on clinical experience, we initially suspected that the patient had infectious keratitis, possibly caused by a viral or bacterial infection. Eye drops for antibacterial and antiviral treatment were administered to the patient. However, two weeks later, the corneal ulcer progressed and there was hypopyon. IVCM findings indicated sparse subepithelial nerve fibers, with most regions lacking nerve presence. Additionally, DC density increased from 41.80 ± 26.13 n/frame to 88.60 ± 20.55 n/frame within two weeks (Table 2).

Given the progressive corneal ulceration and hypopyon, she underwent ocular debridement of the right eye and anterior chamber paracentesis for microbiological diagnostics. The anterior chamber was irrigated, and aqueous humor samples were collected for high-throughput sequencing. Corneal exudate culture results were negative and no pathogenic microorganisms were identified through sequencing analysis. Comprehensive autoimmune serology showed normal levels of autoimmune markers and antibodies. Based on the characteristic clinical manifestations, response to treatment, and temporal association with confirmed SARS-CoV-2 infection, a presumptive diagnosis of COVID-19-related NK was established, representing a virus-related keratopathy.

### Protracted neurotrophic keratopathy following SARS-CoV-2 infection

Management of COVID-19-related NK comprised: (1) antiviral agents (e.g., ganciclovir) to mitigate herpesvirus reactivation risk potentiated by SARS-CoV-2-induced immune dysregulation; (2) nocturnal antibiotic ointment/gel for corneal exposure protection and infection prophylaxis; (3) nutritional supplementation; (4) glucocorticoids for inflammation control; (5) artificial tears; (6) 5 mg/mL tropicamide phenylephrine eye drops for anterior chamber inflammation prevention; and (7) two AMTs (Fig. 1). During a severe inflammatory phase (approximately 2 months), 1% prednisolone acetate ophthalmic suspension superseded 0.1% fluorometholone therapy. Four months later, the hypopyon subsided, and the lesion became localized but remained Mackie stage III (Fig. 1). Five months later, central tarsorrhaphy was performed. She did not come for follow-up for a year due to personal excuses. Twenty months after SARS-CoV-2 infection, she showed up and reported alleviation of ocular symptoms. Examination revealed the transparent cornea with only residual corneal macula (Fig. 2). Corneal sensation increased to 30–40/60. During the subsequent follow-up, the patient’s condition remained stable and the NK healed, so lysis of tarsorrhaphy was performed.

**Fig. 3:**
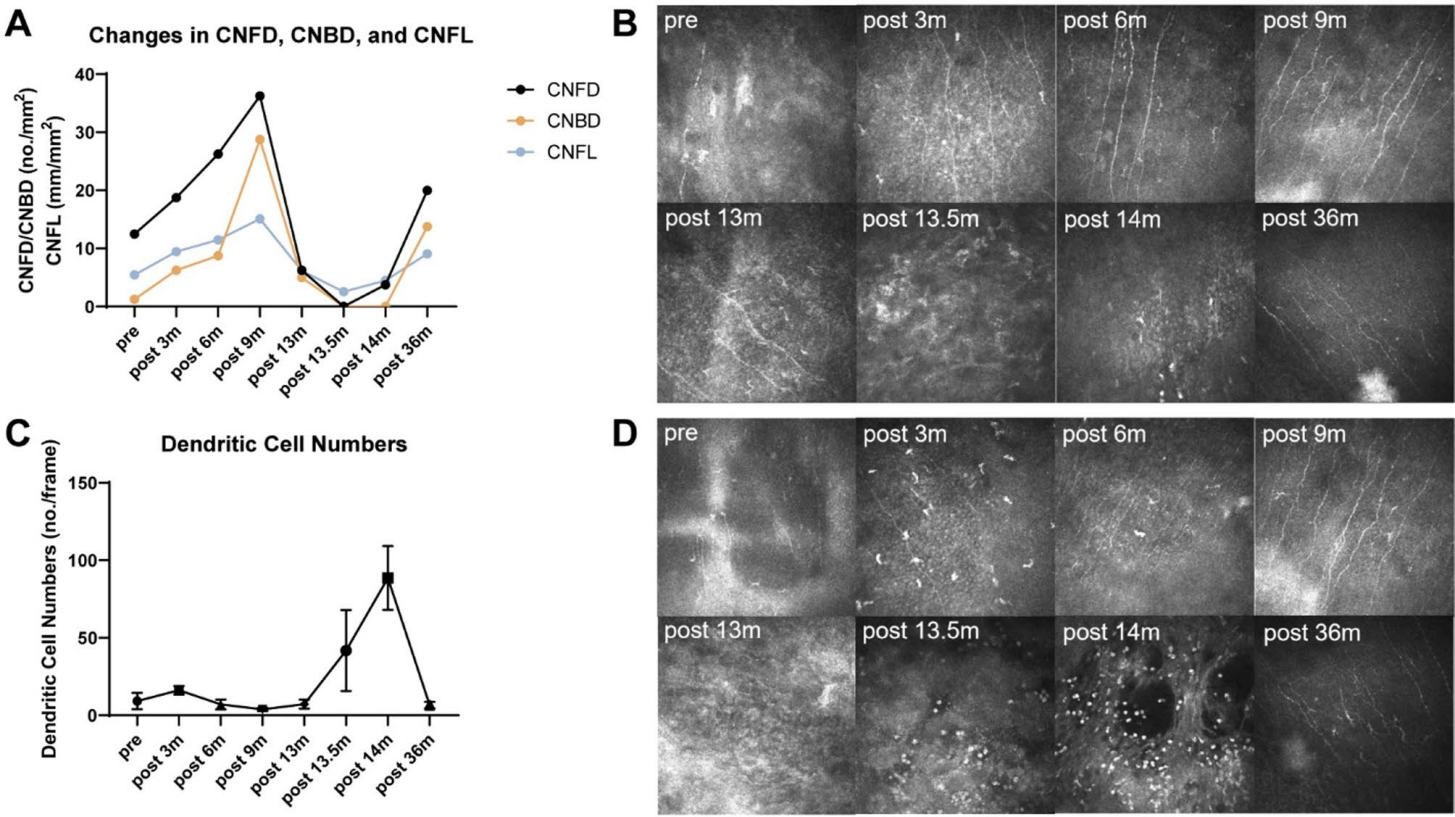
In vivo confocal microscopy outcomes of the patient. **A** Changes in CNFD, CNBD, and CNFL of the patient. **B** In vivo confocal microscopy images of corneal nerves. At 36 months postoperatively, the area with the highest density of corneal nerves was not captured during in vivo confocal microscopy examination due to the patient’s right-sided tarsorrhaphy. **C** Changes in corneal dendritic cell numbers of the patient. (D) In vivo confocal microscopy images of corneal dendritic cells. Abbreviations: CNFD, corneal nerve fiber trunk density, the number of fibers per mm^2^ (no./mm^2^); CNBD, corneal nerve branch density, the number of branch points on the main fibers per mm^2^ (no./mm^2^)

Reviewing the treatment process, the patient’s corneal sensation exhibited a trajectory characterized by complete loss, partial recovery, relapse, and eventual partial recovery. Corneal nerve innervation shifted from denervation to neurotization, reverted to denervation following viral infection, and ultimately returned to neurotization (Fig. 3 A, B). DC density initially increased, then decreased, and subsequently rose again post-viral infection (Fig. 3 C, D). DCs played dual roles: facilitating corneal nerve regeneration after MICN and aggravating nerve injury via immune-mediated mechanisms following SARS-CoV-2 infection [4, 14]. Following resolution of recurrent NK after MICN, corneal nerve regeneration was observed, demonstrating that MICN effectively promoted corneal nerve regeneration. Neural pathways reconstructed through peripheral nerve transplantation can regenerate despite prolonged viral infection.

## Discussion

We present a rare case of virus-related keratitis following NK management. To our knowledge, no literature reports describe COVID-19-related keratitis after MICN.

This diagnostic challenge was compounded by: (1) normal autoimmune markers and antibody profiles, thereby minimizing endogenous keratitis probability; (2) IVCM findings excluding fungal (absent hyphae) and Acanthamoeba (no cysts) etiologies, suggesting bacterial or viral pathogenesis; and (3) negative bacterial/fungal/parasitic results from both high-throughput sequencing and corneal exudate cultures. Despite undetected DNA viruses in sequencing analyses, viral etiology remains the most plausible diagnosis, warranting further virological characterization for pathogen identification.

Differential diagnostic considerations distinguishing this presentation from other viral etiologies are summarized in Table 3 [15, 16]. Common DNA viral pathogens–including varicella-zoster virus, cytomegalovirus, Epstein-Barr virus and adenovirus–were excluded based on incongruent medical history and clinical manifestations. Herpes simplex keratitis posed the greatest diagnostic challenge due to overlapping clinical features and absence of definitive biomarkers.

**Table 3:** Differential diagnosis of this case from other viral etiologies

Etiology	Differences from this case
Varicella-zoster keratitis	History of herpes zoster ophthalmicus, with band-like distribution of herpes on unilateral face, and effective treatment with ganciclovir
Cytomegalovirus keratitis	Nummular KPs, enlarged corneal endothelial cells with multinucleated appearance and intranuclear inclusion bodies observed on IVCM examination, and effective treatment with ganciclovir
Epstein-Barr virus keratitis	History of mononucleosis
Epidemic keratoconjunctivitis/Adenovirus keratitis	Bilateral ocular involvement typically without corneal stroma, associated with tender preauricular adenopathy

*Abbreviations*: *KPs *Keratic precipitates

Latent herpes simplex virus (HSV) reactivation may occur secondary to febrile illnesses, corticosteroid administration, or immunosuppressive therapies, potentially triggering recurrent corneal infection. Table 4 details key differential diagnostic criteria between COVID-19-related keratitis and herpes simplex keratitis [14, 15, 17–24]. Three clinical features argue against HSV etiology: (1) no documented herpetic episodes in the medical history; (2) atypical corneal ulcer morphology devoid of classic dendritic or geographic patterns; and (3) absence of multinucleated giant cells on IVCM. Although HSV-induced NK can cause chronic epithelial defects requiring extended therapy, the suboptimal clinical response to empirical anti-HSV treatment further precludes HSV as the primary etiological agent.

**Table 4:** Differential diagnosis between herpes simplex keratitis and COVID-19-related keratitis

	Herpes simplex keratitis	COVID-19-related keratitis
Type of virus	DNA virus	RNA virus
Course of disease	Usually less than a year post-onset, recurrent episodes, ganciclovir treatment being effective, and ulcers healing in 7–10 days	Usually single episode, lesions healing in 5 days to more than 3 months
Past medical history	Probably a history of herpetic ocular disease or labial herpes	SARS-CoV-2 infection
Symptoms and clinical manifestations	Photophobia, epiphora, blepharospasm, decreased corneal sensitivity, and decreased vision	Relatively mild
Corneal lesions	Dendritic or geographic ulcers, stromal opacity or edema, and persistent epithelial defect if leading to NK	Shallow white turbid focus similar to ground glass, corneal phlyctens or diffuse punctate epithelial defects, and stromal opacity or edema
IVCM examination	Multinucleated giant cells and long-term loss of corneal nerve fibers	Long-term loss of corneal nerve fibers, may showing beaded axons and neuroma-like formations of corneal sub-basal plexus

The patient received nine months of topical corticosteroid therapy following MICN, succeeded by a four-month withdrawal period. This extended immunosuppressive interval and subsequent immune reconstitution may have primed aberrant immune responsiveness. During SARS-CoV-2 infection, relative immunosuppression–compounded by potential NK reactivation–likely contributed to accelerated keratitis progression and symptom severity.

However, no published precedents support diagnosing COVID-19 keratitis based solely on temporal association with SARS-CoV-2 infection. Absent direct ocular virological confirmation in this case, a definitive COVID-19 keratitis diagnosis could not be established. Nevertheless, the sequential development of corneal hypoesthesia, ulceration, and hypopyon post-SARS-CoV-2 infection corresponds precisely to Mackie stage III NK manifestations. Thus, COVID-19-related NK remains etiologically plausible. Collectively, given the clinical chronology and exclusion of alternative etiologies, COVID-19-related NK emerges as the most probable diagnosis.

According to established literature, primary ophthalmic symptoms after SARS-CoV-2 infection encompass conjunctival hyperemia, ocular pain, photophobia, and reduced visual acuity [19, 20]. Characteristic clinical presentations typically feature conjunctival congestion or ground-glass-like corneal opacities, with corneal ulceration representing an uncommon clinical manifestation [17–21]. Diagnostic evaluation incorporates conjunctival swab/corneal scraping samples for pathogen detection via polymerase chain reaction (PCR) alongside inflammatory cytokine profiling in ocular secretions, notwithstanding occasional false-negative PCR results [17–19]. Current therapeutic paradigms emphasize localized immunosuppressive, antiviral, and antimicrobial regimens [17, 20, 21]. Studies confirm that SARS-CoV-2-infected patients with neurological manifestations exhibit corneal nerve fiber loss and DC upregulation [14]. Furthermore, 91.31% of convalescent patients demonstrate persistent corneal sub-basal plexus abnormalities–including beaded axons and neuroma-like formations–up to ten months post-recovery, indicating chronic COVID-19-related neuropathy [22].

Divergent neuroinvasive mechanisms between RNA and DNA viruses are exemplified by SARS-CoV-2 and HSV. Neurotropic entry pathways differ fundamentally: SARS-CoV-2 enters the central nervous system via hematogenous dissemination or retrograde axonal transport, infecting ACE2-expressing neurons, glia, and other neural cells to induce neurological sequelae (e.g., headaches, brain fog) [25, 26]. Conversely, HSV establishes latency in trigeminal ganglia and reactivates during immunosuppression, triggering neuroinflammation and herpetic neuralgia [27]. Mechanistically, SARS-CoV-2-mediated neural injury involves immune hyperactivation, neuroinflammatory cascades, and cerebrovascular compromise–albeit without consistent parenchymal histopathology [14, 25, 28, 29]. In contrast, HSV-induced damage primarily stems from ganglionic viral reactivation driving neuroinflammation and neuropathic pain [27].

In this case, resolution of symptoms, restoration of corneal structural integrity and sensation, DC reduction, and increased nerve density collectively demonstrate NK resolution and post-viral neural regeneration. Viral infection may disrupt ocular immune homeostasis, inducing corneal nerve damage and sensory impairment–a potential contributor to postoperative keratitis following MICN that delays sensory recovery. Critically, the eventual restoration of corneal sensation and NK remission confirms both neural pathway regeneration and MICN’s therapeutic efficacy. This clinical trajectory–incorporating disease course, interventions, and outcome–suggests that peripherally reconstructed neural pathways retain regenerative capacity despite prolonged viral insult. Thus, MICN demonstrates significant utility in refractory NK management.

This study has several limitations. Pandemic-related constraints in our institutional setting precluded timely corneal scraping samples or aqueous humor PCR analysis. Direct detection of SARS-CoV-2 RNA in ocular tissues would have provided definitive etiological confirmation.

## Conclusions

We reported a rare case of COVID-19-related keratitis following corneal neurotization. The protracted clinical course underscores that post-MICN keratitis management in COVID-19 patients may constitute a prolonged therapeutic challenge requiring extended multidisciplinary intervention. Following a two-year clinical course, the patient eventually attained recovery of corneal sensation and evidence of reinnervation, indicating the regenerative capacity of neural pathways reconstructed via MICN despite severe viral insult.

## Supplementary Information

Supplementary Material 1.

## Data Availability

No datasets were generated or analysed during the current study.

## Abbreviations

NK: Neurotrophic keratopathy
MICN: Minimally invasive corneal neurotization
BCVA: Best-corrected visual acuity
IVCM: in vivo confocal microscopy
DCN: Direct corneal neurotization
AMT: Amniotic membrane transplantation
DC: Dendritic cell
HSV: Herpes simplex virus
bFGF: basic fibroblast growth factor
CNFD: Corneal nerve fiber trunk density, the number of fibers per mm^2^ (no./mm^2^)
CNBD: Corneal nerve branch density, the number of branch points on the main fibers per mm^2^ (no./mm^2^)
CNFL: Corneal nerve fiber length, the total length of nerves mm per mm^2^ (mm/mm^2^)
HSV: Herpes simplex virus
PCR: Polymerase chain reaction
