# Supplementary material for: Corneal neurotization meets COVID-19: a case report of minimally invasive corneal neurotization complicated by COVID-19-related keratitis
Source: J Ophthalmic Inflamm Infect. 2025 Aug 9;15:60. doi: 10.1186/s12348-025-00521-6 (PMC12335426; doi:10.1186/s12348-025-00521-6)
Supplement: Supplementary file 1 — Supplementary Material 1. [file 12348_2025_521_MOESM1_ESM.docx]

**Supplemental Table 1.** Changes in CNFD and CNBD of the patient.

| **Variables** | **pre** | **post 3m** | **post 6m** | **post 9m** | **post 13m** | **post 13.5m** | **post 14m** | **post 36m** |
| --- | --- | --- | --- | --- | --- | --- | --- | --- |
| CNFD (no./mm^2^) | 12.50±9.88 | 18.75±4.41 | 26.25±11.18 | 36.25±6.85 | 6.25±4.41 | 0.00±0.00 | 3.75±3.42 | 20.00±10.27 |
| CNBD (no./mm^2^) | 1.25±2.79 | 6.25±6.25 | 8.75±5.59 | 28.75±14.39 | 5.00±5.23 | 0.00±0.00 | 0.00±0.00 | 13.75±17.34 |

**Abbreviations:** CNFD, corneal nerve fiber trunk density, the number of fibers per mm^2^ (no./mm^2^); CNBD, corneal nerve branch density, the number of branch points on the main fibers per mm^2^ (no./mm^2^).
